# Gastric Dilation due to a Neuroleptic Agent in an Elderly Patient: A Case Report

**DOI:** 10.1155/2014/961048

**Published:** 2014-08-05

**Authors:** V. Parent, L. Popitean, A. Loctin, A. Camus, P. Manckoundia

**Affiliations:** ^1^Service de Médecine Interne Gériatrie, Hôpital de Jour, Hôpital de Champmaillot CHU, BP 87909 2, rue Jules Violle 21079, Dijon Cedex, France; ^2^INSERM/U1093 Motricité-Plasticité: Performance, Dysfonctionnement, Vieillissement et Technologies d'Optimisation, Faculté des Sciences du Sport, Université de Bourgogne, 21078 Dijon, France

## Abstract

Neuroleptics may cause side effects, some of which are little known. We describe here a case of gastric dilation related to treatment with a neuroleptic in an elderly man. To our knowledge, such a case has never been reported in the literature. A 76-year-old man, living in a nursing home, was hospitalized for general weakness and abdominal pain. He had dementia with behavioral disorders treated with cyamemazine, a sedative and anxiolytic neuroleptic. Given a clinical suspicion of intestinal occlusion, an abdominopelvic computerized tomography scan was performed before the patient was admitted to our hospital. This computerized tomography scan did not show intestinal occlusion and there was no mention of gastric dilation in the computerized tomography scan report. Thus, acute gastroenteritis was suspected. The usual medications were stopped and symptomatic treatment for gastroenteritis was started. Quickly, his clinical state and biological parameters returned to normal and his usual treatment, including cyamemazine, was started again. The next day, the digestive symptoms, except for obstipation, reappeared. The abdominal X-ray showed gastric dilation without intestinal occlusion. The neuroleptic was stopped again and symptoms vanished the next day. This report underlines all of the necessary precautions and surveillance around drug prescription, especially in elderly persons.

## 1. Introduction

Because elderly people often suffer from several comorbidities, they take various drugs simultaneously. Neuroleptics are among the drugs most often prescribed in order to treat behavioral disorders due to dementia syndrome, delirium, or other conditions. Neuroleptics, however, may cause side effects, some of which are little known. We describe here a case of gastric dilation related to treatment with a neuroleptic in an elderly person. To our knowledge, such a case has never been reported in the literature.

## 2. Case Report

**Figure 1:**
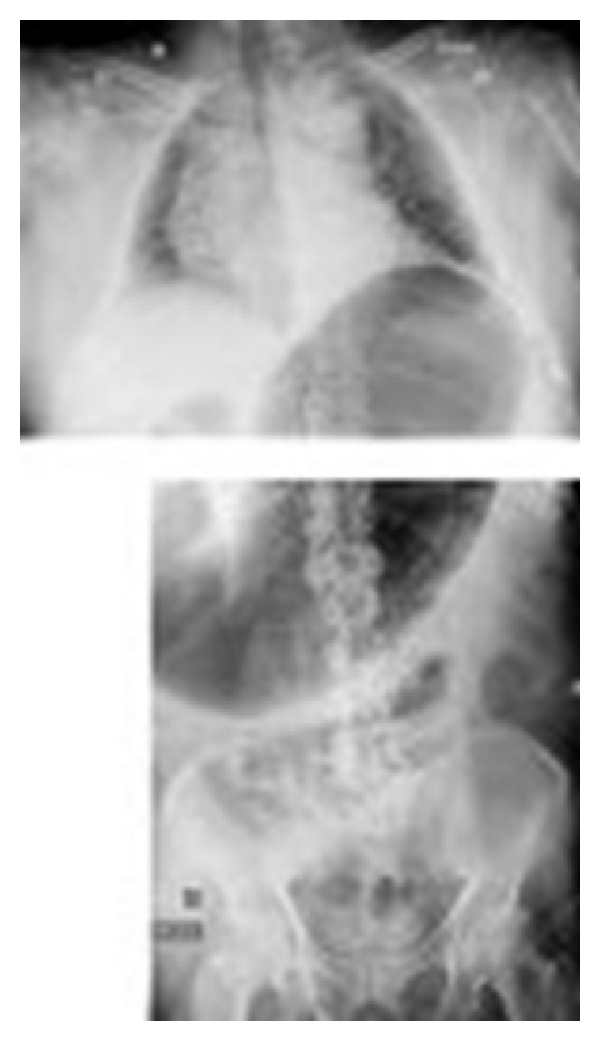
The first abdominal X-ray showing considerable gastric dilation.

**Figure 2:**
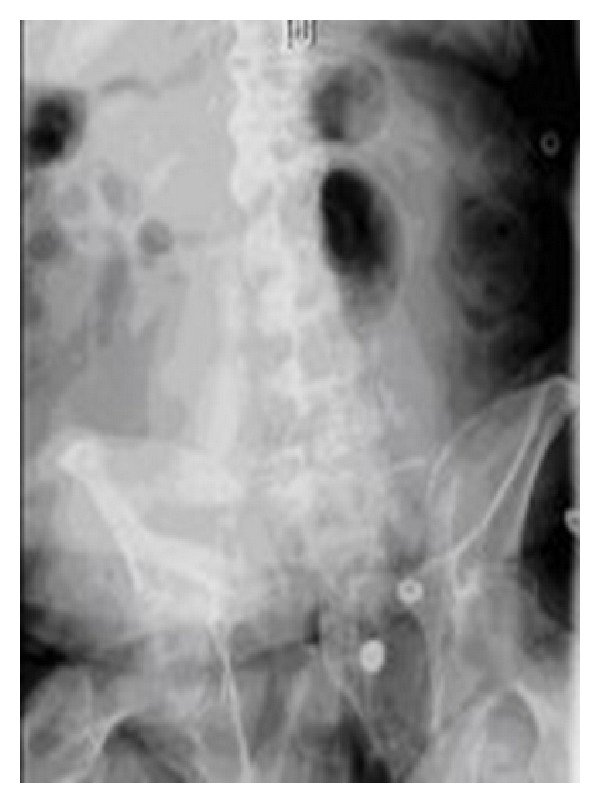
The second abdominal X-ray, performed after the neuroleptic was stopped the second time leading to the disappearance of the digestive symptoms and the return to a normal stomach size.

A 76-year-old man, living in a nursing home, was hospitalized in a geriatric unit for suspected intestinal occlusion. His medical history consisted of heart failure, hypertension, chronic renal insufficiency, type 2 diabetes, dementia with behavioral disorders, prostate adenoma, dyslipidemia, hepatic steatosis, and sleep apnea syndrome. He had been treated with cyamemazine, a sedative and anxiolytic antipsychotic drug, for several years and was also taking hydroxyzine, insulin, tamsulosin, and atorvastatin. The patient presented digestive disorders including uncontrollable food and fecaloid vomiting, diffuse abdominal pain, and obstipation, all of which appeared suddenly. The clinical examination showed general weakness without fever, and the abdominal examination found a reduction in bowel sounds at the auscultation and confirmed diffuse abdominal pain. Initial biological screening showed an increase in C-reactive protein at 40 mg/L (normal < 3.2), neutrophils at 10000 cells/mm^3^ (normal 1800–7500), urea at 8.1 mmol/L (normal 2.1–7.1), and creatinine at 118 *μ*mol/L (normal 53–115) (estimated creatinine clearance 55 mL/min/1.73 m^2^). In addition, aspartate aminotransferase and alanine aminotransferase were slightly elevated. Capillary glycemia was between 9.7 and 17.7 mmol/L, and glycosylated hemoglobin stood at 8.1%, confirming slightly to moderately uncontrolled diabetes. The report of the abdominopelvic computer tomography (CT) scan, performed in an emergency before the patient was admitted to our hospital, did not confirm the intestinal occlusion and made no mention of gastric dilation. Thus, the diagnosis of acute gastroenteritis was raised. His usual medications were stopped and a symptomatic treatment for gastroenteritis, which included intravenous hydration, paracetamol, fasting, and gastric aspiration, was started. Quickly, the general weakness, vomiting, and diffuse abdominal pain vanished with normalization of intestinal transit and biological parameters. Thus, his usual treatment, including cyamemazine, was reintroduced. The next day, all of the digestive symptoms described above, except obstipation, reappeared. The abdomen was distended and tympanic. The abdominal X-ray showed considerable gastric dilation without intestinal occlusion (Figure 1). The neuroleptic (cyamemazine) was stopped again and the symptoms vanished the next day. A new abdominal X-ray showed a normal size of the stomach (Figure 2). A few days later, the patient returned to his nursing home.

## 3. Discussion

Neuroleptics, which are often used in the elderly in certain neurological and psychiatric affections such as dementia with cognitive disorders, are known to induce intestinal side effects [1]. These intestinal side effects include constipation, paralytic ileus, acute megacolon (Ogilvie syndrome), intestinal perforation, intestinal necrosis, and ischemic colitis [2–5]. Gastrointestinal effects are also possible in the context of neuroleptic malignant syndrome because of the autonomic nervous system disorders associated with this syndrome. However, to our knowledge, no cases of gastric dilation have been described in the literature so far. Only one case of gastrointestinal hypomobility with intestinal dilatation has been reported [2, 6]. In the case we report here, the neuroleptic was cyamemazine, which is a sedative antipsychotic drug with a strong anxiolytic action. Cyamemazine behaves as an antagonist at both 5-hydroxytryptophan_2c_ (5-HT_2c_) receptors and to a lesser extent at 5-hydroxytryptophan_3_ (5-HT_3_) receptors. These 5-HT_2c_ and 5-HT_3_ antagonistic actions of cyamemazine, which involve blockade of these receptors, can explain, at least in part, its beneficial therapeutic actions on anxiety [7–9]. In our report, the causal relationship between cyamemazine and the gastric dilation was proven by the reintroduction of the neuroleptic drug after a period of withdrawal. Reintroducing cyamemazine resulted in the reappearance of the gastrointestinal symptoms. In addition, the gastric dilation seen on the abdominal X-ray performed during the treatment with the neuroleptic vanished when the drug was stopped. We regret the absence of abdominopelvic CT scan pictures and the fact that gastric dilation was not mentioned in the CT scan exam report. It is possible that the CT scan was centered on the bowels (small bowel and colon) in an emergency in order to eliminate intestinal occlusion and any gastric dilation, if present, would not have been seen.

Diabetes, which can induce gastroparesis [10] principally through dysautonomia [11], may have contributed to the occurrence of gastric dilation. Gastroparesis can cause delayed gastric emptying [11] and lead to gastric dilation without mechanical obstruction. However, this hypothesis was ruled out given the evolution of the patient's clinical and paraclinical signs; indeed, the gastric dilation vanished once the drug was stopped.

A reassessment of all medications, including those taken for several years, is imperative. Indeed, some adverse effects occur after several years of good tolerance to the drug as in the case reported here.

The present case report brings to the attention of the medical community a previously unknown adverse effect of neuroleptics. Moreover, it shows all the necessary precautions and surveillance that need to be exercised around the prescription of drugs, especially in elderly people. The physician must be vigilant for signs of adverse reactions and envisage the withdrawal of the suspect drug even if it has been taken for several years with no side effects.
